# A Survey on Routing Protocols for Large-Scale Wireless Sensor Networks

**DOI:** 10.3390/s110403498

**Published:** 2011-03-24

**Authors:** Changle Li, Hanxiao Zhang, Binbin Hao, Jiandong Li

**Affiliations:** State Key Laboratory of Integrated Service Networks, Xidian University, Xi’an, Shaanxi 710071, China; E-Mails: zhx0930@163.com (H.Z.); dreamfly_99@126.com (B.H.); jdli@mail.xidian.edu.cn (J.L.)

**Keywords:** large-scale wireless sensor networks, scalability, routing protocol, survey

## Abstract

With the advances in micro-electronics, wireless sensor devices have been made much smaller and more integrated, and large-scale wireless sensor networks (WSNs) based the cooperation among the significant amount of nodes have become a hot topic. “Large-scale” means mainly large area or high density of a network. Accordingly the routing protocols must scale well to the network scope extension and node density increases. A sensor node is normally energy-limited and cannot be recharged, and thus its energy consumption has a quite significant effect on the scalability of the protocol. To the best of our knowledge, currently the mainstream methods to solve the energy problem in large-scale WSNs are the hierarchical routing protocols. In a hierarchical routing protocol, all the nodes are divided into several groups with different assignment levels. The nodes within the high level are responsible for data aggregation and management work, and the low level nodes for sensing their surroundings and collecting information. The hierarchical routing protocols are proved to be more energy-efficient than flat ones in which all the nodes play the same role, especially in terms of the data aggregation and the flooding of the control packets. With focus on the hierarchical structure, in this paper we provide an insight into routing protocols designed specifically for large-scale WSNs. According to the different objectives, the protocols are generally classified based on different criteria such as control overhead reduction, energy consumption mitigation and energy balance. In order to gain a comprehensive understanding of each protocol, we highlight their innovative ideas, describe the underlying principles in detail and analyze their advantages and disadvantages. Moreover a comparison of each routing protocol is conducted to demonstrate the differences between the protocols in terms of message complexity, memory requirements, localization, data aggregation, clustering manner and other metrics. Finally some open issues in routing protocol design in large-scale wireless sensor networks and conclusions are proposed.

## 1. Introduction

Recent advances in micro-electro-mechanical systems and low power and highly integrated digital electronics have led to the development of micro-sensors. As the cost of the individual sensors has been reduced, it has become feasible to deploy large numbers of sensors in a relevant region, constituting large-scale wireless sensor networks (WSNs). In general, the application scenarios of a WSN include target field imaging, intrusion detection, weather monitoring, security and tactical surveillance, distributed computing, detecting ambient conditions such as temperature, movement, sound, light, or the presence of certain objects, inventory control, and disaster management [1]. Large-scale deployment of the nodes can increase the accuracy of the information and enhance the scope for detection, and so on. Therefore research focusing on large-scale WSNs has attracted much more attention.

Compared with normal *ad hoc* networks, there are some special considerations concerning routing protocol design for WSNs. First of all, because the individual sensor devices have limited power and battery replacement or recharging is typically not practical, any routing protocol must work in an energy-efficient manner. In addition, the nodes in the network are always randomly deployed and the position information is not available without a Global Positioning System (GPS) service for the sake of economic cost reduction. Especially in large-scale WSNs where the numbers of nodes can reach thousands or even more, the scalability objective of the routing protocol to handle the long distance which the sensed data must travel from sensors to collection nodes and the huge amount of network overhead must be taken into consideration.

Normally, according to the underlying network structure, the traditional WSNs routing protocols fall into three classes known as flat, hierarchical and location-based [1]. In flat networks, all the nodes play the same role and coordinate to relay the sensed packets to specific sink nodes. The routing protocols belonging in this category include Sensor Protocols for Information via Negotiation (SPIN [2,3]), Directed Diffusion (DD [4]), Rumor Routing [5], Gradient-based routing (GBR [6]), Energy-Aware Routing (EAR [7]), and the Minimum Cost Forwarding Algorithm (MCFA [8]), *etc*. In hierarchical networks, all the nodes are divided into several groups with different responsibility levels. The high level nodes are responsible for aggregation and some management work, and the low level nodes for sensing the surroundings and collecting information. There are also plenty of routing protocols in this hierarchical family, such as Low Energy Adaptive Clustering Hierarchy (LEACH [9]), Threshold-Sensitive Energy Efficient Sensor Network Protocol (TEEN [10]), Minimum Energy Communication Network (MECN [11]), Self-Organizing Protocol (SOP [12]), Sensor aggregates routing [13], Virtual Grid Architecture routing (VGA [14]), and Hierarchical Power-Aware Routing (HPAR [15]), *etc*. Location-based protocols utilize positional information to relay data to some desired regions rather the whole network, while additional hardware devices for acquiring the location of other nodes is indispensable. The protocols falling into this part include Geographic Adaptive Fidelity (GAF [16]), Geographic and Energy Aware Routing (GEAR [17]), Greedy Other Adaptive Face Routing (GOAFR [18]), and Span [19], *etc*.

In the literature there are numerous and rich works surveying the routing protocols for WSNs from different points of view and with different concerns. They all analyze the strengths and weaknesses of the respective routing protocols, but none of the papers has focused on the scalability objective of the protocols especially designed for large-scale WSNs. For instance, Al-Karaki *et al*. in [1] presented a comprehensive survey of routing techniques which are classified based on the network structure and protocol operation respectively, and outlined challenges and future research directions in this aspect. Luo *et al*. provided in [20] an overview of existing routing protocols that support data fusion in wireless sensor networks. They categorized the algorithms as routing-driven, coding-driven and fusion-driven, depending on their design principles. Alwan *et al.* in [21] overviewed fault tolerant routing techniques in WSNs, classifying them into two main schemes: retransmission based and replication based. It should be noted that clustering is an elegant method for grouping sensor nodes, meanwhile making data aggregation feasible and more efficient. An example of this method would be the aforementioned LEACH. The authors in [22,23] classified the hierarchical protocols according to the objectives, the desired cluster properties and the clustering process. Again the papers reviewed the general protocols for WSNs, but not differentiating them for large-scale scenarios or not. In fact, all the papers summarized and analyzed the routing protocols with different requirements, for instance to prolong the network lifetime, to balance energy consumption, to reduce overall network overhead *etc.* based on the large deployment of the sensor nodes. To the best of our knowledge, the work presented in this paper is the first attempt at a comprehensive survey with focus on the scalability of the routing protocols. Hence, in this paper we will give an insight into the hierarchical protocols designed especially for large-scale WSNs and compare their advantages and disadvantages in metrics like message complexity, memory requirement, cluster formation and maintenance, data aggregation, energy consumption, network lifetime, end-to-end delay *etc.* for extending network scale. We categorize them according to their design objective as control overhead reduction, energy consumption mitigation and energy balance, with the goal of increasing energy efficiency.

In this paper we present a survey of recent advances in routing protocols for large-scale WSNs, our aim is to provide a full understanding of research challenges in the emerging protocols. The rest of the paper is organized as follows: in Section 2, a detailed analysis of currently innovative protocols for large-scale WSNs is presented, with the objective of highlighting the critical factors influencing protocol design. Section 3 summarizes the characteristics of these protocols and compares them and we present the related open issues for the hierarchical routing protocol design. Finally, we conclude with final remarks in Section 4.

## 2. Routing Protocols in Large-Scale WSNs

**Figure 1.**
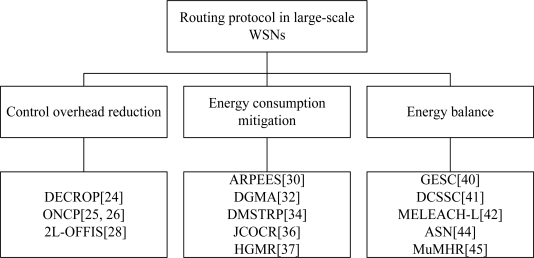
Routing protocols in large-scale WSNs: a taxonomy.

We discuss first the state-of-the-art routing protocols for large-scale WSNs. Due to the particularities of a large-scale WSN, how to enhance the energy efficiency is a problem of great significance. We summarize the methods for improving energy efficiency such as control overhead reduction, energy consumption mitigation and energy balance according to their motivation. The classification is shown in Figure 1.

*Control overhead reduction-based category*: such routing protocols aim to reduce the control overhead to enhance the energy efficiency with the goal of extending network longevity. They use innovative designs to simplify the route construction process other methods to substitute the routing process, thus the control overhead can be reduced.

*Energy consumption mitigation-based category*: the routing protocols in this class aim to mitigate the energy consumption. They exploit various means to achieve this target, such as dynamic event clustering, multi-hop communication, cooperative communication and so on. These methods can consume the energy appropriately and avoid wasted energy.

*Energy balance-based category*: in this class, the routing protocols are proposed from different points of view, but with a uniform objective which is energy balance. When a node is assigned some redundant and repetitive missions what has been assigned to other nodes, the node will consume energy disproportionally and become quickly useless. It appears that energy balance-based methods can also improve the energy efficiency of the sensor nodes.

In the remainder of this section we elaborate the above classes of routing protocols by providing an overview of various algorithms proposed in the literature under each category.

### 2.1. Control Overhead Reduction Algorithms

***DECROP***. A simple but efficient routing protocol named Distributed and Effective Cluster Routing Protocol (DECROP) is proposed in [24] with the purpose of decreasing the number of control messages, shortening the average end-to-end delay and satisfying other requirements such as data aggregation *etc*. DECROP includes three processes: initialization with distributed cluster formation, data transmission and route maintenance.

**Figure 2.**
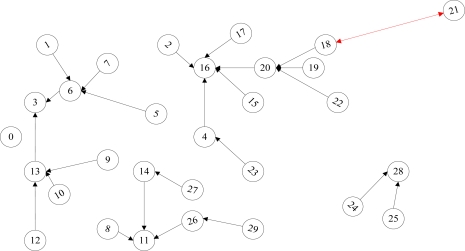
Distributed cluster forming process in DECROP.

During the initialization period, a cluster is formed simultaneously to aggregate data packets from cluster members and to reduce transmission power during the delivery to the base station (BS). The initialization aims at making each sensor confirm its neighbor nodes and the pre-hop node along the path to the BS which is node 0 in Figure 2. Initially the BS broadcasts an initialization message. The node receiving the message for the first time takes the transmitter as the pre-hop node, and renews the transmitting ID in the message with its own ID and rebroadcasts the message. Then the receiver will ignore the subsequent messages. In the end, all the nodes build the forwarding path as Figure 2 shows. During the initialization and after collecting its neighbor information, the local sensor will announce itself as cluster head (CH) by broadcasting a declaration message when its total neighbor count reaches *N*. *N* is a network parameter associated with communication radius and nodes deployment. The one-hop neighbors start to join the cluster by sending request messages and the two-hop neighbors have to resort to the one-hop neighbors by delivering request messages. Therefore, the clusters are created in two hops instead of the club structure (one hop). It is possible that some nodes are far away from the cluster head and have not joined any cluster. As shown in Figure 2, the red double-head arrow represents that node 21 is a single node that has not joined any cluster.

During data transmission, the cluster head aggregates data packets from the cluster members, tags the packets with cluster head information, and delivers them to the pre-hop nodes which are confirmed during initialization process. During the delivery of the packets, the intermediate nodes could record the path backwards to the specific cluster head. By this way, it is convenient to route packets from the BS to the destinations according to its cluster head information. As a special case shown in Figure 2, node 21 is sending its data packets directly to the pre-hop node instead of any cluster head. When these packets arrive at a cluster head, node 21 will be incorporated in its cluster. If some links are broken, the route maintenance process is triggered. The downstream node will broadcast an error message including the unreachable pre-hop node and its hop count towards the BS. One of the receivers will reply the message and act as the new pre-hop node if the unreachable node is not its own pre-hop node and its hop count towards the BS is less than that recorded in the error message.

After the initialization process, all the nodes will have constructed the forwarding path thus saving a large amount of time and overhead for building routes. The adoption of the cluster model enables the data aggregation. In the cluster, the nodes are organized by two hops instead of the conventional club way (such as the single-hop communication in LEACH [9]) and the amount of clusters is reduced accordingly. However, when the cluster is larger, the energy consumption of the cluster head is increased considerably. Another disadvantage is that the tree route makes the nodes closer to the BS consume energy faster which will reduce the overall network lifespan.

**Figure 3.**
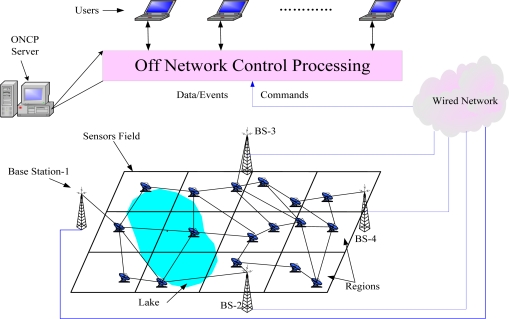
Network and application model of ONCP.

***ONCP***. Wu *et al.* in [25,26] proposed a routing solution called Off-Network Control Processing (ONCP) that achieves control scalability in large-scale sensor networks by handing over certain amount of routing functions to an “off-network” server. The function of the ONCP server is to compute the “coarse grain” global routing, which consists of a sequence of regions. During the delivery of sensing task along the global routing, a “fine grain” local routing is performed by the local sensor nodes. By this tiered routing approach, wide dissemination of network control messages is avoided. As depicted in Figure 3, the sensing area is pre-partitioned into regions, in which each sensor node maintains a never changed region ID.

The nodes periodically update the ONCP server with information about the residual energy in the region and the inter-connectivity metrics between regions. The latter is defined as the residual energy of the sensor nodes having direct connection with the ones in the neighboring region. Based on these updates, the server is able to compute the most energy-optimal global routes from each region to an appropriate base station upon receiving sensing requests from users. It should be noted that the global route consists of a sequence of regions from the source region to an appropriate base station. The sensing task request is source routed to the desired region using the region-level global route and the local routes computed on-demand during the propagation. Then the target sensors start generating data at the specified rate, and send the data to the appropriate base station along the global route and the local routes outlined above in the reverse order.

Min-hop routing and MaxMin routing [27] are used to compute the global route in order to minimize the end-to-end energy consumption and evenly distribute the energy consumption loads on regions to avoid traffic hot-spots. For a given base station and target sensing region, first the MaxMin value of paths is found, and then the smallest hop-counts path among them is chosen. During the construction of local routes, clustering is adopted as a technique to avoid redundant broadcasts and too much overhead introduction. A cluster head originates and broadcasts a local route request message, which contains the originating cluster head ID, the originating region ID, the target region ID, and a hop-count field. After receiving the message from its own region, the cluster head increments the hop-count of the message and re-floods the message. When the message arrives at a neighboring region that is not the target region, the receivers will discard the message. When the message arrives at the target region, the cluster head replies a local route reply message, which is to be forwarded back to the originating cluster head through the reverse pointers set up during the route request message flooding. By this way, the control message is constrained in the sequence regions of the global route.

An advantage of ONCP is that the control overhead incurred during the construction of “fine grain” and local route will not grow exponentially as the network expands by computing the “coarse grain” global route, because the overhead in the area of sequence regions of global route is limited. Therefore ONCP scales well with growing network size. However, its benefits could be sustained only when the overhead of network status update and sensing task dissemination is lower than the control overhead of global route creation message flooding in other competing approaches. In addition the pre-configuration of region ID in each sensor node adds implementation complexity of ONCP.

***2L-OFFIS***. In order to prolong the network lifetime, Jamalipour *et al.* [28] proposed a two-layer OFFIS (2L-OFFIS) based on Optimized Forwarding by Fuzzy Inference System (OFFIS) [29] presented earlier. In 2L-OFFIS, the cluster structure inherited from LEACH is adopted, but with either intra-cluster or inter-cluster multi-hop routing during data transmission. A fuzzy inference system is introduced to consider a collection of metrics such as distance, power and link usage in deriving the optimal path from the source to the destination.

2L-OFFIS includes two parts, which are formation of cluster and data forwarding. In the first phase, the algorithm inherits the feature of LEACH in grouping sensor nodes. That is the nodes choose themselves as the cluster heads based on a pre-defined probability and then the sensors pick up a CH to join the cluster based on the receiving signal strength from the CH. Time division multiple access (TDMA) is used in each cluster when transmitting sensed packets in order to power off the transceiver until the right assigned time slots.

**Figure 4.**
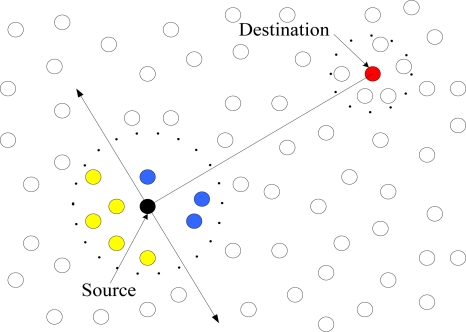
Election of relay nodes in OFFIS.

The only differentiation with LEACH is that in 2L-OFFIS the more distant nodes will get earlier slots and the closer nodes will get later slots. In the second phase, the sensed data will be first delivered to the corresponding CH and then transmitted to the sink node. During the delivery, either intra-cluster or inter-cluster, OFFIS is applied to select the next hop among its neighbors. It works as follows: the forwarding node utilizes its neighbors’ location information to calculate the distance between the node and its neighbors and the distance between its neighbors, and the linear distance between the source and the destination is also required. Besides, the neighbor’s battery usage and link usage are also combined to make a fuzzy inference used to select a neighbor node as the next hop. Generally, the nearest node from the source and from the shortest path, also with the most abundant resource will be selected as the next hop. As shown in Figure 4, blue nodes are the candidate nodes in the forwarding path, and yellow nodes are discarded.

In this protocol, a GPS positioning service or some localization algorithms are assumed to be available. Therefore the routing protocol is more scalable than that without position awareness. The next hop during transmission is chosen independently without route request flooding in the whole network, and so there is no need to maintain the ID of each sensor node. Additionally, every sensor node just needs to maintain the neighbor information, and accordingly the storage costs to store the routing table are saved. In a word, the energy consumption will be reduced thanks to these advantages and the network lifespan will be prolonged. However, the assumption of a GPS positioning service will increase the monetary costs and the multi-hop routing increases the end-to-end delay with respect to the single hop routing used in LEACH.

### 2.2. Energy Consumption Mitigation Algorithms

***ARPEES***. Quang *et al.* in [30] proposed an Adaptive Routing Protocol with Energy efficiency and Event clustering for wireless sensor networks (ARPEES). The main design features of the proposal are energy efficiency, dynamic event clustering, and multi-hop relay considering the trade-off between the residual energy available of the relay nodes and distance from the relay node to the base station. The operation of ARPEES is segmented into rounds, and each round has two stages, *i.e*., forming clusters and selecting cluster heads followed by selecting relay nodes and data transmission.

In the first stage, all the nodes are in a sleep state to save battery power in the beginning. When an event is detected in the network, nodes near the event become activated and start measuring the specific sensed attribute. If the sensed attribute value is greater than a predefined threshold, those nodes form a cluster and broadcast a REQ_CLUSTER [*ID*(*i*),*E_res_*(*i*),*I*(*i*)] message which consists of the node ID, the amount of residual energy and descriptive information of the sensed data about the event to their neighbors. After that the nodes set their timer for *t_1_*. During the period time *t_1_*, each nodes within the cluster executes the Cluster Head function as follows:

(1) $$\begin{array}{c} F_{\mathrm{CH}}\left(i\right)=E_{\mathrm{res}}(i)\times I\left(i\right),\forall i\in X\\ \mathrm{Max}\;F_{\mathrm{CH}}\left(i\right)\overset{\forall i\in X}{\underset{\mathrm{set as}}{\to }}\mathrm{Cluster Head} \end{array}$$

This function ensures that the node which is the nearest to the event and with maximum energy available will be selected as the cluster head. The cluster head stores the node ID of all the nodes in this cluster, and creates the TDMA schedule to arrange each node when the nodes can transmit their sensed data to the cluster head.

In the second stage, using the TDMA schedule described above, each sensor node transmits the sensed information to its cluster head during their allocated transmission period. According to the TDMA scheme, the node that has more data information will transmit with priority and with more time slots than others. Then data aggregation will be performed at the CH and the total bits of data packets can be reduced accordingly. In the phase of selecting relay nodes and creating a route, the cluster head broadcasts an REQ_RELAY message to all the neighboring nodes. Each node that receives the REQ_RELAY message calculates its residual energy and distance to the base station, and then puts the results into an ACK_RELAY message, and sends the message back to the cluster head. The cluster head waits a period for receiving all the ACK_RELAY messages from relay node candidates and checks whether it can transmit data to the base station directly. The desired relay node should satisfy three conditions: the maximum amount of residual energy, the maximum distance from the cluster head and the minimum distance to the base station, being located on the approximate straight path between cluster head and base station. These conditions can be expressed by the Relay Node function, which is defined as follows:

(2) $$\begin{array}{l} F_{\mathrm{RN}}\left(j\right)=E_{\mathrm{Res}}\left(j\right)\times \frac{d\left(\mathrm{CH},j\right)}{d\left(j,\mathrm{BS}\right)}\times \text{cos}\alpha_{j},\forall j\in Y\\ \mathrm{Max}\;F_{\mathrm{RN}}\left(j\right)\overset{\forall j\in Y}{\to }\mathrm{Relay Node}\\ \text{cos}\alpha_{j}=\frac{d{\left(\mathrm{CH},j\right)}^{2}+d{\left(\mathrm{CH},\mathrm{BS}\right)}^{2}-d{\left(j,\mathrm{BS}\right)}^{2}}{2d\left(\mathrm{CH},j\right)d\left(\mathrm{CH},\mathrm{BS}\right)} \end{array}$$

ARPEES outperforms LEACH thanks to its multi-hop transmission, thus balancing energy consumption over several relay nodes rather than focusing energy consumption on the CH. Besides, it achieves load balance by selecting the node with maximum residual energy as the relay node, and the hot spot problem is alleviated.

In order to increase the energy efficiency of ARPEES in a large-scale scenario and balance energy consumption required for sensing data, forming clusters, selecting cluster heads, and relaying data to different sensor nodes to prolong the whole network lifetime, the so-called SC-ARPEES protocol was proposed by Quang *et al.* in [31]. In SC-ARPEES, the processing phases are similar to those in ARPEES, except for an additional phase of removing redundant nodes. SC-ARPEES inherits the advantages of ARPEES, but it may cover a larger area using fewer sensors. In large-scale WSNs, substantial numbers of nodes are deployed randomly over the entire desired area, and the sensing regions of different nodes may partially overlap. Therefore, the network will contain numerous redundant nodes. When the cluster formation phase is done, each non-cluster node will check whether it is redundant by Maximum Sensing Coverage Region (MSCR) algorithm put forward in [31]. If so, then it sends a sleep message to the cluster head, or else it sends an active message to the cluster head and waits for a TDMA schedule message from the cluster head.

The clusters in ARPEES are constructed on demand, which makes the protocol scalable to the network extension. The property whereby a node with the most descriptive event information is selected as a CH node helps the ARPEES protocol reduce the data packets transmitted within a cluster and decrease energy consumption correspondingly. Moreover, data aggregation at CH nodes further reduces transmitted data packets. SC-ARPEES outperforms ARPEES in reducing redundant nodes and thus improves network performance. According to the simulation results presented in [31], SC-ARPEES reduces the average residual energy up to 30% compared with the original ARPEES, because SC-ARPEES achieves energy balance by checking redundant information and scheduling the nodes’ activities. Therefore the network life time is prolonged. However, in order to calculate the distance from the BS, the BS has to broadcast beacon messages periodically with the maximum radio power to cover the whole network field. On the other hand, in every round the CH node has to keep the transceiver active all the time in order to receive packets from cluster member nodes and the possibility of energy exhaustion is not handled.

***DGMA***. In terms of energy consumption reduction and network end-to-end delay decrease, a Data Gathering algorithm based on Mobile Agent (DGMA) for the cluster-based wireless sensor network was proposed in [32]. The region where an emergent event occurs is clustered dynamically based on the event severity, by which the scale and lifetime of clusters are determined. In each cluster a mobile agent is utilized to traverse every member node to collect sensed data. In the higher level of the network, a virtual cluster is formed among the cluster heads and the base station, and multi-hop communication is adopted for sensed data delivery to the base station.

In DGMA, all the sensor nodes are in “restraining” state and they are activated only when some emergent event occurs. Then the nodes having monitored the event are clustered. After the event intension gets reduced, the clustered nodes will change to a “restraining” state for the sake of energy consumption reduction. In the cluster, the tree structure is used to save energy instead of single hop communication between the sensor nodes and the cluster head.

After the cluster construction is complete, a route for the mobile agent, which is equipped on the cluster head, is used to traverse all the member nodes for collecting the sensed event data. This process is started up by the cluster head and repeated at every cluster member by broadcasting a request packet, and anticipating a reply from its each neighbor for getting residual energy, path loss, and event intension information of the neighbor. Then a next hop is calculated by the equation as follows:

(3) $$M=\underset{j\in {\mathrm{FT}}_{i}}{\max}\left(\alpha \times \frac{E_{j}}{E_{\max}}+\beta \times \frac{C_{\max}}{C_{j}}\right)\times \mu \times I_{j}$$

wherein *E_j_* denotes the residual energy node *j*, *C_j_* denotes the path loss, and *I_j_* denotes the event intension and *α*, *β*, *μ* separately denote the force of the residual energy, path loss and stimulated intension for route selection. The above equation means that more residual energy, less path loss and more event intension imply higher probability that the node will become the next hop of node *i*. Then, the mobile agent will move to the next hop for data collection. Due to the limited buffer space in the mobile agent, in this protocol data is fused on those in-between nodes that can not only sense, but also forward data in order to reduce space occupation.

To deliver the sensed data to the final destination (here the base station) in the higher level of the network a virtual cluster is formed wherein the base station acts as the cluster head. As in the local cluster, a multi-hop communication is adopted. The current cluster head will select the node which is the closest to the base station in the neighboring nodes as its next hop. If the distance from all neighbor nodes to the base station is longer than that from the node itself, the node will communicate with the base station directly.

In the simulation part in [32], it was shown that DGMA is more scalable than EDMGP, which was presented in [33]. When the number of the sensor nodes increases, the energy consumption in DGMA increases more slowly. Furthermore, the dynamic cluster formation feature further reduces the energy consumption. The use of a mobile agent reduces energy consumption, but extends the delay for the cluster head to collect all the sensed data from all the member nodes. The chain-like route delivery of data by the cluster head makes the node closest to the base station overloaded and destroys the reliability.

***DMSTRP***. Huang *et al.* proposed a routing protocol named Dynamic Minimal Spanning Tree Routing Protocol (DMSTRP) in [34]. When the network size becomes larger, this scheme outperforms LEACH and Base Station Controlled Dynamic Clustering Protocol (BCDCP [35]) in terms of network lifetime and delay by introducing the concept OF Minimal Spanning Tree (MST) instead of THE clubs which are used in BCDCP to connect nodes in clusters. The main idea of DMSTRP is to use MSTs to replace clubs in two layers of the network: intra-cluster and inter-cluster. Because clubs are less effective than a spanning tree in connecting the nodes if the network area is larger, DMSTRP is an elegant solution in larger network areas.

**Figure 5.**
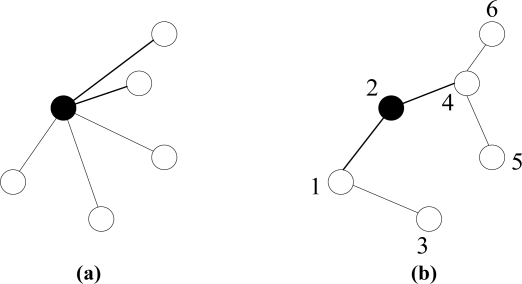
**(a)** A club structure of a cluster in LEACH and BCDCP. **(b)** A MST in DMSTRP.

LEACH chooses clubs as the basic topology of the network, as shown in Figure 5(a), and managing clubs does not need multi-hops and thus makes the routing path simple. One step further in BCDCP, the CHs are connected by a tree instead of a club and the BS functions as the manager of the whole network, so BCDCP is more energy-efficient than LEACH. DMSTRP improves BCDCP further by connecting nodes in clusters by MSTs. In each cluster, all the nodes including the CH are connected by a MST and then the CH acts as the leader to collect data from the nodes on the tree. On the higher level, all the CHs connected by another MST cooperate to route data towards the BS. The data fusion process is handled during the packet transmission along the tree route.

In DMSTRP, the structure of a MST is also utilized to alleviate the collisions among the transmitting nodes. Through reasonably arranging the transmitting sequence for the nodes within an intra-cluster, multiple nodes can transmit messages simultaneously, therefore increasing the throughput. The transmitting scheme is as follows: if a transmitter of a branch does not need to receive any data, this branch can be used to transmit data. However, if a number of son-nodes want to deliver data packets to the same father-node at the same time, only the first node which to be traversed can transmit the data to the father-node, and the others should be waiting for the next round. In a round of communication, if a node faces the situation of transmitting and receiving happening at the same time, receiving has priority.

A comparison is shown in Figure 5. A club structure cluster is shown in Figure 5(a). The structure of DMSTRP and the transmitting sequence in a MST are depicted in Figure 5(b) where the first round transmission queue is {3, 5}, which means node 3 and node 5 can transmit their data simultaneously. The transmitting queue in the following round is {1, 4 and 6}.

Obviously, DMSTRP consumes energy more efficiently than LEACH and BCDCP, because the average transmission distance between nodes is reduced through the multi-hop intra-cluster and inter-cluster communications, and thus the energy dissipation of transmitting data is potentially reduced. Furthermore, due to the reasonable schedule, the transmission collision is alleviated and DMSTRP can achieve shorter delay compared with LEACH and BCDCP. But the transmission schedule creates more overhead.

***JCOCR***. Ge *et al.* in [36] proposed a novel idea by introducing cooperative communication from a mobile *ad hoc* network (MANET) to a WSN for energy reduction in such energy constrained networks. In the first stage during packet delivery, the coalition head broadcasts data packets to all the nodes within its coalition; in the second stage the coalition head, together with the nodes in the coalition, cooperatively forward the packet to the next hop destination. The procedure lasts until the packet reaches its final destination. A larger coalition would reduce the cooperative cost, but may require more multicast energy to reach nodes located further away. Whereas a smaller coalition would require less multicast energy, it would have higher cooperative costs. The authors aimed to find the optimal coalition size to minimize the total transmission cost. During the one-hop delivery of packets, the optimal coalition size is derived by:

(4) $$\begin{array}{l} C_{\mathrm{ab}}=\underset{k_{a}}{\min}\left[P_{k_{a}}^{M}+P_{k_{a}}^{C}\right]\\ P_{k_{a}}^{M}=\max\left\{P_{a1\_\mathrm{direct}},P_{a2\_\mathrm{direct}},\cdot \cdot \cdot P_{{\mathrm{ak}}_{a}\_\mathrm{direct}}\right\}\\ \underset{P}{\min}P_{k_{a}}^{C}=P_{a}+\sum_{j}^{k_{a}}P_{j}\\ s.t.\;\;\;P\leq P_{\max}\\ \;\frac{1}{N}{\left(\sum_{j=1}^{k_{a}}\sqrt{P_{j}/d_{\mathrm{ab}}^{\beta }}+\sqrt{P_{a}/d_{\mathrm{ab}}^{\beta }}\right)}^{2}\geq \gamma \end{array}$$

In the equations above, $P_{k_{a}}^{M}$ indicates the multicast cost to reach *k_a_* neighbors, $P_{k_{a}}^{M}$ means the cooperative cost from the *k_a_* (plus node a itself) nodes to node b and *P_ai_direct_* is the cost of point-to-point transmission from node a to node i. $P_{k_{a}}^{M}$ takes the maximum value among the set of *P_ai_direct_* that aims to be able to reach the farthest node in the coalition. The second restriction is intended to assure the receiver could receive the packets successfully by combining the signals from the coalition nodes.

**Figure 6.**
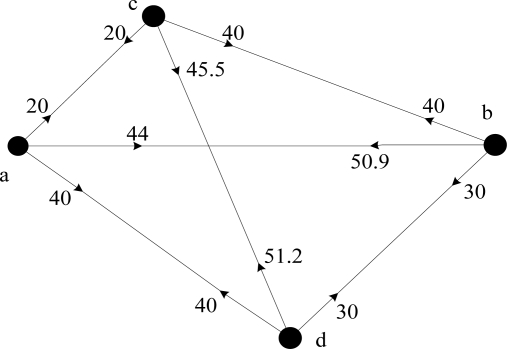
4-node cooperative routing graph in JCOCR.

After finding the optimal coalition size, the original network could be modeled as an edge-weighted and directed graph, as Figure 6 shows. In general the number of neighbor nodes is a key factor, because the node with more neighbors could transmit with lower cost. Therefore, *C_ab_* might not be equal to *C_ba_*. Based on the directed graph, the routing problem from the source node to the destination node is formulated as shortest path routing problem and could be solved by Dijkstra’s algorithm to find the minimal energy consumption route. Moreover, if the routing path is required to satisfy the delay constraint, the problem boils down to a Delay Constraint Least Cost (DCLC) problem which is NP-hard. Throughput was also considered as additional QoS requirement when route searching in [36]. However, the more requirements during route searching, the more complex the algorithm will become.

JCOCR is proven to be more energy-efficient than cooperative geographic routing [2] for the following reasons: it exploits power allocation during cooperative forwarding; it optimizes the coalition size to minimize energy consumption; and it chooses the routing path based on global information instead of the local information, which means it chooses the minimum sum of costs along the path instead of minimizing the one-hop cost. JCOCR may easily consider more QoS requirements, but more requirements will add the complexity of the algorithm and limit its applications. Apart from this, the complexity is closely related with nodes density. The coalition size is optimized at individual nodes and the routing graph is visualized and constructed at every single node so that the more neighbors the local node has, the more calculations it has to do for optimizing the coalition size. In addition the geographic information which is desirable for the optimization of coalition size represents an extra cost.

***HGMR***. Hierarchical Geographic Multicast Routing (HGMR) for wireless sensor networks was proposed in [37] with the aim of enhancing data forwarding efficiency and increasing the scalability to a large-scale network. HGMR seamlessly incorporates the key design concepts of the Geographic Multicast Routing (GMR) [38] and Hierarchical Rendezvous Point Multicast (HRPM) protocols [39], and optimizes the two routing protocols in the wireless sensor network environment. HGMR starts with a hierarchical decomposition of a multicast group into subgroup of manageable size using HRPM’s key concept of mobile geographic hashing. Within each subgroup, HGMR uses GMR’s local multicast scheme to forward a data packet along multiple branches of the multicast tree in one transmission.

In HGMR, the multicast group is divided into subgroups using the mobile geographic hashing idea: the deployment area is recursively partitioned into *d^2^* equal-sized square sub-domains called cells, where *d* is decomposition index depending on the encoding overhead constraints, and each cell consists of a manageably-sized subgroup of members. An Access Point (AP) is responsible for all members in its cell, and APs are managed in turn by a Rendezvous Point (RP). The role of each AP or RP is mapped to some unique geographic location by a simple hash function. The node that is currently closest to that location then serves the role of AP/RP, and routing to the AP/RP is conveniently achieved by geographic routing. To join a hierarchically decomposed multicast group, a node first hashes the multicast group identifier (GID) to obtain the hashed location of the RP via a hashed function and sends a JOIN message to the RP, which is the same as in the flat domain scenario. After receiving the value of the current *d* of the hierarchy from the RP, the node utilizes the hash function with *d* and the node’s location to compute the hashed location of the AP belonging to its cell. Note that computing the hashed location assumes that all nodes know the approximate geographic boundaries of the network. After that the source builds an overly tree, the *Source* → *APs* tree, whose the vertices are active APs in a topology graph; and an *AP* → *Member*s overly tree is also built from the AP, considering each member as the vertex.

When a source needs to send data packets, it utilizes the unicast-based forwarding strategy belonging to HRPM to propagate data packets to each AP along the *Source* → *APs* tree. In each cell, adjusting the value of *d*, the number of members for which an AP is responsible does not increase too much. Therefore, GMR’s cost over progress optimizing the broadcast algorithm, which is used to select the next relay node at each hop, contributes to reduce the number of data transmissions while maintaining a low encoding overhead compared with the unicast communication. Sensor nodes running GMR use the position of their neighbors to select the subgroup which is the best one to deliver the message towards the destination, and the selected neighbors can reduce most the total route to destination. When no neighbor of the current node can reduce the route to the destination, face routing is used to circuitously search the path to the destination.

In HGMR, the geographic hashing algorithm makes the membership management very simple with almost zero cost. According to the number of the nodes which play the different roles, HGMR selects the transmission methods for different hierarchies in reason, which makes the routing energy-efficient and scalable. However, the RP is in charge of too much missions in HGMR, which may bring the problem of rapid energy consumption and make the entire network collapse.

### 2.3. Energy Balance Algorithms

***GESC***. In [40] the authors proposed an energy-efficient distributed clustering protocol, named Geodesic Sensor Clustering (GESC). GESC aims to prolong the network lifetime by distributing energy consumption evenly, considering the localized network structure and the remaining energy of neighboring nodes.

One of the main parts of the protocol is the estimation of the significance of the sensors relative to the network topology. The significance is calculated in the view of the local network at individual nodes. That means the significance of the same node is distinct respect to different local nodes. The view of the local network is defined as the sub-network associated with the set of vertices in *N_12_* (*v*) which is the combined set of one-hop neighbors and two-hop neighbors of node *v*. And the node significance index *NI* (*v*) is calculated by:

(5) $$\mathrm{NI}\left(v\right)=\sum_{u\neq v\neq w\in V}\frac{\sigma_{\mathrm{uw}}\left(v\right)}{\sigma_{\mathrm{uw}}}$$

The denominator denotes the number of the minimum hop paths from *u*∈ V to *w*∈ V and the numerator denotes the number of the minimum hop paths from *u* to *w* that some vertex *v*∈ V lies on. Larger values for the *NI* index of a node *v* indicate that node *v* can reach others on relatively short paths. The author has made some improvements in the calculation and achieved a complexity of *O*(n*m) for a network with *n* vertices and *m* edges.

The clustering protocol is divided into rounds which are composed of a clustering formation procedure (CFP) and a network operation procedure (NOP), taking up time *T_CFP_* and *T_NOP_*, respectively. CFP clustering is triggered to select new cluster heads. At the start, nodes exchange “Hello” messages which contain the list of their neighbors and their residual energy (*E_residual_*) with their neighbors. By this means, every individual node is aware of the existence of its two-hop neighbors. Then the following detailed phases will be carried out:

1. In this phase, after collecting one-hop and two-hop neighbors of node *v*, node *v* executes *CalculateNodeImportanceIndex* over its two-hop neighborhood.
2. Then node *v* runs a sorting algorithm to obtain a list of its neighbors, sorted in descending order of their *NI* (*v*).
3. Examine one-by-one the members of the list obtained in phase 2. If the currently examined one-hop neighbor *u* covers at least a two-hop neighbor, then designate the one-hop neighbor *u* as candidate cluster head node.
4. Obtain a list of its candidate cluster head nodes by sorting them based on *E_residual_*. And choose the node with maximum residual energy as the cluster head. On the occasion of the same residual energy, select the minimum set of one-hop neighbors that cover the two-hop neighborhood.

During the first round, all four phases are executed, and after that only phase 4 will be executed until a neighboring node dies, given that the topology will never change due to the immobility scenario. After the network clustering process, each node transmits the sensed data to its cluster head and likewise the cluster head transmit it to its cluster head until the data reaches the information sink. Data aggregation is performed at cluster heads whenever they receive the data from neighboring nodes. The current cluster head will select as next cluster heads only those significant nodes that cover the two-hop neighborhood which is uncovered yet. The current cluster head delivers the message to the next hop after making sure there are not any one-hop neighbors who have already broadcast the message, otherwise it discards the message. When the energy consumed is more than 99.99% of its initial energy level, the node considers itself “DEAD” and transmits a “DEAD” message to one-hop neighbors, which will delete the dead node from the list and those two-hop neighbors is covered only by the dead node. Finally, they execute the CFP in order to elect new cluster heads.

In GESC, the cluster head is elected depending on the location of the source, the residual energy and the importance of the candidate, thus avoiding the effect of “hot-spots”. Additionally, the energy consumption is distributed over all the sensors because cluster heads is elected independently at individual nodes and differs at each time and at each node, by this means prolonging the network lifetime. However, all the phases will be performed whenever a node failure occurs, which is prone to produce large overhead.

***DCSSC***. In order to utilize the spatial correlation among the sensed data, reference [41] proposed Distributed Clustering Scheme based Spatial Correlation (DCSSC) for grouping sensors based on similarity of data readings. The sensor nodes that have the highest similarity in observations are grouped into the same cluster, and accordingly, they can be scheduled to alternatively report their sensed data for energy saving. Additionally, a dynamic backbone is constructed for efficient data collection. The dissimilarity is defined to describe the degree of difference between the data readings of any two sensor nodes. It is calculated by:

(6) $$d(s,v)=\omega_{1}\left|s_{1}-v_{1}\right|+...+\omega_{n}\left|s_{n}-v_{n}\right|$$

where *s_i_* and *v_i_* indicates some types of reading data at the respective node and *ω_i_* denotes how much it affects relative part.

The cluster construction is started with a Cluster Formation (CFRM) message broadcasted from the sink. Upon receiving the message, each initial (INI) node will change state to gateway-ready (GWR) and set two timers, which are *t_req_* for transmitting Cluster head Request (CHREQ) message and *t_wait_* for receiving Cluster head Advertisement (CHADV) message. When the former timer expires, a CHREQ message is sent out. Upon receiving the message every INI node calculates the dissimilarity measure, and will change to cluster head candidate (CHC) if they are proved to be strongly correlated (meaning *d*(*s*, *v*) ≤ *Ã*, the latter part is a predefined value), setting a timer *t_adv_* at the same time. Then the new CHC node declares itself as a CH node by broadcasting CHADV message when the timer *t_adv_* expires. After receiving the CHADV message, every temporary state node, INI or CHC node here, calculates the dissimilarity measure with the new CH. If they are strongly correlated, the receiver will become a member (MEM) of the cluster; otherwise, it goes to GWR state. The GWR node upon receiving the message needs to check the predecessor ID (p-ID) of the message with its own. If they are matched, GWR will enter GW state; otherwise, it becomes MEM of the cluster. However, if until *t_wait_* expires the GWR node has not got any CHADV messages, it will change state to CHC. Then the GW and MEM nodes continue to propagate the CFRM message by creating and broadcasting cluster-extend (CEXT) message to discover the rest of the network, and the receiver, if strongly correlated with the originating CH, will become a MEM node of this cluster and the message disseminator becomes the cluster-extend node. The process described above proceeds until the entire sensor nodes are grouped into respective clusters.

It should be noted that this scheme constructs a dynamic backbone as an accessory of cluster formation based on the reversed paths of the cluster formation message propagation paths, and no extra overhead is incurred. The backbone consists of CH, GW, and CEXT nodes which are responsible for collecting the sensor reading data and propagating the control messages from the sink to the entire WSN. During the operation of this scheme, the CH could decide whether its cluster should be split or not when it detects the dissimilarity among sensor nodes is enlarged. If it is, the GW of the cluster will initiate a new local cluster construction phase to regroup these sensor nodes into several clusters. To avoid the existence of too many clusters in the network, the sink node can re-cluster the whole network when the current number of cluster becomes significantly larger than the number of clusters at the previous network-wide clustering.

The obvious advantages of DCSSC is that it groups the sensor nodes with strong correlations into the same cluster and rotates them in turn to wake up to work, and as a result much energy is saved and the energy consumption is balanced. Additionally, this scheme builds clusters once and maintains them on demand instead of refreshing them periodically. The on-demand property reduces by a large amount of overhead for re-clustering all the sensor nodes. However, the spatial correlation degree is defined by the users through a dissimilarity threshold *Ã*, and accordingly the accuracy of the collected data readings from the rotated sensor nodes is discounted. On the other hand, the energy dissipation at the CH node, as an important metric for evaluating performances and extending lifetime of large-scale networks, is not considered.

***MELEACH-L***. Based on LEACH and More Energy-efficient LEACH (MELEACH [42]), Chen *et al.* in [43] proposed an expanded routing protocol, called More Energy-efficient LEACH for large-scale WSNs (MELEACH-L). MELEACH-L makes the major functions of MELEACH applicable to large-scale WSNs. Through controlling the size of each cluster and separating the cluster heads from the backbone nodes, MELEACH-L solves the problems of the channel assignment among neighbor clusters and the cooperation among cluster heads during data collection.

**Figure 7.**
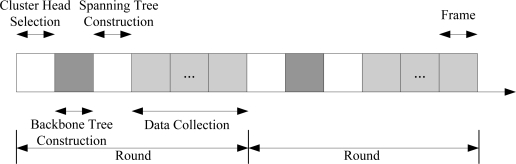
Time-line of MELEACH-L.

The procedure of MELEACH-L is divided into rounds. Each round consists of sequentially tetradic phases: the Cluster Head Selection, the Backbone Tree Construction, the Spanning Tree Construction and the Data Collection. And the time-line of the procedure is shown in Figure 7.

In MELEACH-L, the transceiver of each sensor node is assumed to switch its channel among a set of 20 channels, which range from Channel 0 to Channel 19. Channel 0 is used as the common channel, on which every sensor node and the BS work in the beginning of every round. The detailed procedures may be listed as follows:

1. Cluster Head Selection. First of all, sensor nodes initialize the channel set *C_i_*= {1, 2 … 19}, and set up a timer *T_i_*. When *T_i_* expires, node *i* becomes a CH and broadcasts an Advertisement Message (ADV_i_), including the node ID and its geographical coordinate and the serial number of the intra-cluster channel used in the cluster that node *i* dominates, with the maximum power of its radio. Eventually, if a node *j* hears ADV_i_ and the distance between *i* and *j* is greater than *R/2* (*R* is the maximum transmission radius of the radio), *j* will not join the cluster and delete the channel *i* from *C_j_*; or else, *j* clears *T_j_* and becomes a non-CH node.
2. Backbone Tree Construction. Based on the Energy-aware Virtual Backbone Tree (EVBT) algorithm, some of non-CH nodes are selected to construct an EVBT. The sink node initiates an EVBT Construction Request (ECR) at the beginning of the procedure. The EVBT grows from the sink and spans the entire sensor network. The EVBT algorithm aims to build an energy efficient tree throughout the network. It has three characteristic objectives: the energy consumption for data delivery along the EVBT is minimized; the energy consumption for the EVBT construction is minimized; the tree nodes should have high energy levels.
3. Spanning Tree Construction. When the backbone tree is constructed, each CH selects the closest EVBT node as its upstream node according to the geographical coordinate information. Each client node, which is the node neither a CH nor an EVBT node, chooses the closest CH as its leader according to the information in the ADV messages.
4. Data Collection. Following the TDMA schedule, each cluster member carries out collection and transmission of messages in corresponding time slots. In the schedule, the time slot when a sensor node *i* transmits the aggregated data to its parent node is always after the slots when the children of *i* transmit aggregated data to *i*.

In MELEACH-L, the massively redundant nodes are utilized to deliver the data messages, and that can alleviate CHs’ energy consumption. The adoption of EVBT further improves the balanced energy consumption, and also make the network lifetime become much longer. Moreover the interference restraints between clusters which are working on different channels increase the network throughput. However, the utilization of multiple channels increases economic cost taking much frequency spectrum, and the cluster, the backbone tree and the spanning tree must be rebuilt periodically due to the assumption that every node must know the coordinate information of the entire network. The former makes the protocol uneconomical and the latter incurs much overhead.

***ASN***. There is not a definite method which can be used to address the problem of communication between the CH and the BS in LEACH or other protocols based on LEACH. Most of LEACH-alike protocols assume that a CH can communicate directly with the BS, but it is always not the case. Consequently a multi-hop transmission scheme is utilized in [44]. In order to alleviate the effect of self-induced black hole, an alternative sensor nodes (ASN) is also proposed in this work. The operation of this routing algorithm is separated into rounds. Each round includes a set-up phase for constructing clusters and a steady-state for transmitting data from sensor nodes to the BS via CHs. To be more specific, the algorithm is divided into five steps for completing the process of communication in every round:

1. Counting hop number. At the beginning of communication, the BS broadcasts a message including hop-count parameter, and then the nodes which receive the message forward it to their neighbors until every node receives the message. When the broadcasted messages are received by a sensor node through different paths, the sensor node caches all the routes towards the BS into the routing table in the memory space.
2. Hierarchicalizing sensor nodes. In order to hierarchicalize sensor nodes into different layers to set the level of sensor nodes, the sensor nodes which communicate with the BS via the same hop-count are set into the same layer.
3. Clustering in the system. Each node uses a value within 0 and 1 to compare with a threshold *T*(*n*) which is a function related with the desired percentage of CH nodes during a round. The value has been selected at the start of the round. The node becomes a CH autonomously when its value is smaller than *T*(*n*), and then the node broadcasts an announcement message to its neighbors. Based on the received signal strength form the CH, other non-cluster nodes decide which cluster to join. Additionally, some specially located sensor nodes may not be able to find a cluster in their transmission range, and thus they elect themselves as CH nodes.
4. Transmission and scheduling in a cluster. A CH node schedules all the sensor nodes in its cluster with TDMA scheme to avoid collision.
5. Selecting transmission routes. There are numerous routes from each node to the BS, and the routes with the least hops will be selected for each node. But there may be many routes with the minimum hops. In this case the route with the highest value of the lowest node’s energy parameter will be selected from these routes. Eventually, the search of the alternative sensor node and the data transmission is proceeding at the same time. If a relay node *a* finds a node *b* in its transmission range *R* and the distance between them is smaller than *R/2* and the residual energy of node *a* is smaller than node *b*, node *b* will become an ASN and take the role of the relay node *a*.

By using ASN to balance the loads of the sensor nodes which are close to the BS, the effect of self-induced black holes is greatly alleviated. This protocol is devised based on LEACH, so it also inherits the advantages of LEACH, for example the TDMA scheme can reduce the inter-cluster and intra-cluster collisions. However, a large overhead is generated due to the clustering of every round. The replacement of relay nodes by ASN accompanies the data transmission whenever possible, so the frequent switching of nodes also incurs numerous overhead.

***MuMHR***. Hammoudeh *et al.* in [45] proposed a robust and efficient routing protocol named Multi-path Multi-hop Hierarchical Routing (MuMHR). This protocol is superior to LEACH in terms of load balance and reliability. In order to prevent energy depletion resulting from the same path being constantly occupied for transmission or particular nodes being cluster-heads for a long duration, traffic multiplexing over multiple paths of network level and periodical rotation of the cluster-heads of cluster level were proposed. The operation of MuMHR can be divided into two stages: set-up and data transmission.

During the set-up stage, cluster heads are elected and clusters are created. The sink selects 5% of all the nodes as cluster heads stochastically and broadcasts this information by a discovery message. Every node which has received the discovery message changes its state from “waiting” to “discovered”, and checks whether it has been elected as cluster head or not. If it is, it broadcasts an advertisement message for forming a new cluster. Otherwise, it forwards this message to its neighbors. It is worth noting that every node regards the node from which it received the discovery message as the upstream node along the path nearest to the sink. This path will be used only when the cluster failure occurs. When a node has joined a cluster, it will ignore any other advertisement messages. Moreover, if the back-off waiting timer is activated, the node will choose a cluster head with which the node is separated with the minimum hop. Then based on the residual energy, the node calculates a value indicating its desire to be a cluster-head in the next cluster set-up stage, and inserts the value in the registration packet that the node sends back to the chosen cluster-head. Afterwards the cluster head chooses the highest value and appends its corresponding sender into the cluster-head backup list and registers the node as a member of the cluster, and repeats the selection process among the residual nodes until the cluster round time is ending. When the cluster round time is over, the current CH node flooding an announcement message for renouncing the CH role. Then the node which is the first in the backup nodes list substitutes the CH role after that it receives an announcement message, and has no use for further communication. The CH role will also be handed over to the backup node when a failure occurs in the current cluster-head node.

During the data transmission stage, the non-cluster head nodes transmit sensed messages to their CHs by TDMA schedule. The CHs aggregate the received data, and then deliver the aggregation packet to the sink. The communication of each cluster using different code division multiple access (CDMA) codes to avoid interference with traffic generated by other clusters.

The back-off waiting time gives more time to receive a smaller number-of-hops value, and the minimum hop-count method for choosing the CH nodes shortens the path and also makes the nodes within any clusters be balanced. The application of backup CHs improves the robustness and reliability of network. The back-off waiting timer can reduce broadcast message further, whereas it leads to much more delay. Another weakness is that numerous overhead will be generated due to the construction of cluster which is executed every round.

## 3. Comparison among the Routing Protocols and Open Issues

Our survey shows that each of the various routing protocols has its own strengths and weaknesses, the chief reason being that the design of protocols depends mainly on the different objectives. We summarize recent results on routing protocols in large-scale WSNs in Table 1. The table shows how different routing protocols fall under different categories, and also compares them according to different metrics. A brief explanation for these metrics follows:

**Message Complexity**. An inevitable consideration in the scalability of routing protocols is the complexity properties of routing protocols. Especially message complexity, which represents the number of the exchanged messages needed for route discovery, plays a significant role in the assessment of the scalability of routing protocols. In general, the total number of messages exchanged for route discovery depends on the overall network size, such as the total number of the nodes in the network or the total diameter (in terms of number of wireless hops) of the network. For instance, *O*(*n*) describes the message complexity when each node has to rebroadcast a packet, and the complexity $O\left(\sqrt{n}\right)$ represents that a particular or several routing path are followed. A polynomial *O*(*n*) is related to parameter *n* representing the number of the nodes in the network, and that means the polynomial is linear with the network size. However, to the best of our knowledge, the existing formally analyzed routing protocols do not scale well with the total network size. There is a protocol named cluster overlay broadcast (COB) [46] used in mobile *ad-hoc* networks (MANETs); its message complexity is quadratic in the shortest source-destination distance and independent of the total number of nodes in the network, and this protocol is proved more scalable in large-scale network. Although COB was originally applied for MANET, it was extended to the scenario of WSNs [47]. Reducing the message complexity and overhead, this heuristic idea deserves to be considered in the routing protocol design for large-scale WSNs.

**Memory Requirement**. The memory requirements of the whole network depend on whether each node has to store some data or routing information, such as the data packets which are waiting to be forwarded, neighbor information, cluster information, route information and so on. This can be represented by a polynomial which is related to the parameter *n* concerning the number of the nodes. For instance, if each node has to store its neighbor information, the memory requirement can be described by *O*(*n*). Please note that the result of the memory requirement represents the worst network case discussed in this paper. For instance, a method of event-based clustering is proposed in ARPEES [30] and this method requires the nodes nearby the event store their neighbor information, we assume that the events occurs in the whole network, and thus all the nodes of the network need to store the neighbor information instead of particular nodes. With the network density enhancing caused by the increase of the network size, the nodes need to store many more information. Due to the limited memory capacity of the large-scale WSNs, however, how to efficiently utilize these storage resources is of great significance for enhancing the scalability of the routing protocols.

**Localization**. Position information is of great help to enhance the accuracy and the efficiency of routing protocols, and generally this information can be acquired by GPS. In 2L-OFFIS [28], the nodes can get the position information, and that makes the directed transmission substitute for broadcast communication of the control packet. Therefore the control overhead is decreased. However, the utilization of GPS increases the economic costs, which makes the use of GPS in large-scale deployment of sensors impractical.

**Data Aggregation**. The advantage of hierarchical networks over flat networks is apparent, because in the former network data aggregation could be conducted at cluster head nodes. These nodes collect the sensed messages from its member nodes, and remove the redundant part, thus reducing the total messages towards the sink nodes. By this means, the network energy efficiency is improved.

**Clustering Manner**. “Proactive” means that the clustering of the network is operating before the network operates. Because the clustering is carried out in the entire network and it needs a long time to maintain, it will create more energy cost than “reactive” clustering which is triggered on demand, such as the occurrence of some event. In some emergent cases, the performance of “reactive” routing is not time-sensitive enough.

**Intra-cluster Topology**. In a cluster, the single hop topology can reduce the end-to-end delay to a certain degree, whereas a significant advantage of the multi-hop topology is energy-efficiency. Especially in DMSTRP [34], the topology of the spanning tree, which consists of the multi-hop structure, not only reduces the transmission energy through decreasing the average transmission distance, but also alleviates the collisions in clusters with a schedule scheme utilizing the tree structure.

**Cluster Head Election**. According to the different objectives of each protocol, these protocols have different ways of electing the cluster heads. In ONCP, for instance, “residual energy” is chosen as the criteria to select cluster head to ensure that the cluster head has enough residual energy to process and deliver data packets. That makes the nodes energy-balanced to a certain degree.

**Multi-Path Routing**. Multi-path routing means the traffic is delivered along several paths in order to balance the energy consumption of sensors along the single path. By this method, the data packets could still be delivered successfully in the case of path failure, thus ensuring the reliable delivery of packets. However, a deficiency is that much more overhead may be incurred owing to several sensor nodes must be selected as the next hops.

In hierarchical routing protocols, some sensor nodes are grouped to efficiently relay the sensed data to the sink. The cluster-head plays the specialized role of performing data aggregation and sending it to the sink on behalf the nodes within its cluster. Thus, how to form the cluster is a more interesting and essential research issue concerning such protocols so that the energy consumption and various communication metrics such as latency are optimized. In addition, due to the number of sensor nodes is substantially increased in large-scale WSNs, the nodes nearby the sink will assume more data forwarding tasks so the energy of these nodes is depleted rapidly. That makes the hierarchical routing protocol design challenging.

According to the discussion of the routing protocols for large-scale WSNs in Section 2, it can be concluded that the flooding is usually used for route discovery, route maintenance and topology update in most of the routing protocols mentioned. In large-scale WSNs, this flooding causes such excessive message collisions that the network efficiency is reduced. However, the flooding has obvious advantages over the location-based unicast/multicast in complexity and economic cost without additional equipment such as GPS. Therefore, research on flooding technique is necessary. For instance, an efficient flooding scheme using 1-hop neighbor information in an *ad hoc* network was proposed in [48]. In this scheme, one-hop neighbor information can be obtained by exchanging the HELLO messages in the MAC layer. By choosing the minimum forwarding nodes, redundant flooding messages are reduced. Additionally the connected dominating set (CDS) [49] technique can be also utilized for reducing the redundant flooding messages. Because blind flooding problem also exists in a large-scale wireless sensor network, these efficient flooding schemes are worthy of implementation.

In a large-scale WSN, the deployment of the sensor nodes is dense, and the topology of the network communication is self-organizing and dynamical. Contrary to a wired network, a wireless sensor network does not have a practical backbone structure, and thus the overall nodes in the network must be responsible for routing processes and maintenance of the routing information. The protocols based the diffusion mechanism of the whole network will sharply reduce the utilization efficiency of network resource. This problem will become more obvious in large-scale WSNs. A typical solution is the virtual backbone network routing technique. For instance, a protocol named clique clustering (CC) for backbone formation is proposed in [51], which aims to efficiently deal with those network dynamics that are typical of large-scale WSNs. Through the backbone network, some suitable sub-networks are chosen for constructing communication network, and the backbone nodes belonging to the sub-network are used to maintain routing information and capture the topology construction of the whole network. These behaviors aim to reduce the routing overhead and save network resources at utmost, and adapt the route changes which come from the energy depletion of the nodes. According to the discussion of the characteristics of the routing protocols in large-scale WSNs, there exist open issues which are worth focusing on.

- Through making the complexity of the routing protocol reduced or not related to the network size, the routing protocol will appear to be much more scalable.
- The hierarchical routing protocol is a mainstream method to solve the scalability problem of large-scale networks, but the factors affecting the cluster formation and cluster-head communication are worth reconsidering in future.
- An efficient flooding scheme is challenging in large-scale WSNs.
- The virtual backbone technique can efficiently enhance the utilization of the network resource, which deserves to be further investigated.

## 4. Conclusions

At present routing in large-scale WSNs is a hot research topic, with a limited but rapidly growing set of efforts being published. In this paper we have conducted a comprehensive survey of the various routing protocols in large-scale WSNs, which is the first attempt in the area. We categorized the routing protocols as control overhead reduction, energy consumption mitigation and energy balance ones, depending on their design objectives. We presented a comparison of the routing protocols discussed in the work in terms of message complexity, memory requirement, localization, data aggregation, clustering manner, intra-cluster topology, cluster head selection and multi-path routing. Through these metrics, the reasonable explanations of their strengths and weaknesses were given.

Although the performances of these protocols are encouraging for improving scalability of large-scale WSNs, some issues remain to be considered. First of all, as the number of nodes in large-scale WSNs increases, the density of the network is increased. Therefore, more redundant information is created and this makes the network congestion more serious. On the other hand, in some inclement and unstable environments, a certain degree of redundancy may be desirable to provide the network with reliability. A trade-off between the redundancy reduction and the redundancy utilization is challenging. In addition, data transmission delays are an unavoidable problem when time-sensitive tasks such as fire alarms are assigned to an entire network. In this case, routing must be prepared in advance and maintained constantly. Embedding this consideration in the routing design is desirable. Furthermore, in a large-scale network, communication links become longer and the deployment of the nodes becomes denser. The possibility of link-failure becomes more frequent [52]. Work towards developing techniques for quickly re-establishing valid routes is likely to be of higher importance for improving the robustness of large-scale wireless sensor networks.

Further research should consider other network performance criteria such as the quality of service (QoS) issues posed by the use of video and imaging sensors for the real-time applications, and node mobility in some special environments. Nonetheless, with the increasing functionalities available to a wireless sensor node, more complicated tasks which involve more energy consumption and network overhead may be assigned to the sensor nodes, so how to increase energy efficiency and scalability of the network remains a challenging research area.

## Figures and Tables

**Table 1.** Comparison of different routing protocols.

	**Classification**	**Message Complexity**	**Memory Requirement**	**Localization**	**Data Aggregation**	**Clustering Manner**	**Intra-Cluster Topology**	**Cluster Head Election**	**Multi-path routing**
DECROP [24]	control overhead reduction	*O*(*n*)^2^	Low *O*(*n*)^7^	NO	YES	proactive	multi-hop	node’s degree	NO
ONCP [25,26]	control overhead reduction	*O*(*n*)	Low *O*(*n*)^7^	NO	NO	reactive	single hop	residual energy	NO
2L-OFFIS [28]	control overhead reduction	*O*(*n*)^2^	Low *O*(*n*)^8,^^10^	YES	YES	proactive	multi-hop	random	NO
ARPEES [30]	energy consumption mitigation	*O*(*n*)^2^	Low *O*(*n*)^1^	NO	YES	reactive	single hop	residual energy, information quantity	NO
DGMA [32]	energy consumption mitigation	*O*(*n*)	Low *O*(*n*)^1,^^5^	YES	YES	reactive	multi-hop	event severity	NO
DMSTRP [34]	energy consumption mitigation	*O*(*nlog n*)^3^	Low *O*(*n*)^9^	NO	YES	proactive	multi-hop	random	NO
JCOCR [36]	energy consumption mitigation	$O\left(\sqrt{n}\right)$^5^	Low *O*(*n*)^1^	YES	NO	reactive	single hop	source-based	NO
HGMR [37]	energy consumption mitigation	$O\left(\sqrt{n}\right)$^5^	Medium *O*(n*g)^4^	YES	NO	proactive	multi-hop	encoding overhead	NO
GESC [40]	energy balance	*O*(n*m)	Low *O*(*n*)^1^	NO	YES	reactive	single hop	node importance, residual energy	NO
DCSSC [41]	energy balance	*O*(*n*)^2^	Low *O*(*n*)^10^	NO	YES	proactive	multi-hop	residual energy	NO
MELEACH-L [42]	energy balance	*O*(*nlog n*)^3^	Low *O*(*n*)^5^	YES	YES	proactive	single hop	residual energy	NO
ASN [44]	energy balance	*O*(*n*)^2^	Low *O*(*n*)^8^	NO	YES	proactive	single hop	the number of required CH	NO
MuMHR [45]	energy balance	*O*(*n*)^2^	Low *O*(*n*)^1^	NO	YES	proactive	multi-hop	random	YES

*n* = number of network nodes; *g* = number of the clusters; *m* = number of the edges.

1 To store neighbor information.

2 Flooding-based.

3 The construction of a minimum spanning tree [50].

4 GPS-multicast.

5 Depends on unicast routing protocol.

6 *O*(n * g) if group information is maintained on each node.

7 To store the pre-hop information to the base station.

8 To store the routes information to base-station.

9 To store the link-state.

10 To store the cluster-head information.

Low- The polynomial is linear with the network size, such as *O*(*n*); Medium- The polynomial is quadratic in the network size, such as *O*(n * g) where parameter *g* indicates the number of the clusters and is related to the network size.
